# Federated artificial intelligence monitoring service (FAMOS): an in silico feasibility study

**DOI:** 10.1093/bjrai/ubag016

**Published:** 2026-09-21

**Authors:** Aysha Luis, Andrew Scarsbrook, Mariusz Grzeda, Jesus Perdomo Lampignano, Matt Clark, Bob Wheller, Jack Baldwin, James Cairns, Simran Dhesi, Mark Hall, David J Lowe, Haris Shuaib

**Affiliations:** Newton’s Tree, London, W1B 1NT, United Kingdom; Radiology, Barts Health NHS Trust, London, E1 2ES, United Kingdom; King’s College London, London, SE1 7EH, United Kingdom; Radiology.Leeds Teaching Hospitals NHS Trust, Leeds, LS9 7TF, United Kingdom; University of Leeds, LeedsLS2 9JT, United Kingdom; University of Bristol, Bristol, BS8 1QU, United Kingdom; NHS Greater Glasgow and Clyde, Glasgow, G3 8SJ, United Kingdom; Health Tech Innovation and Translation Lab, University of Glasgow, Glasgow, G3 8SJ, United Kingdom; Radiology.Leeds Teaching Hospitals NHS Trust, Leeds, LS9 7TF, United Kingdom; Radiology.Leeds Teaching Hospitals NHS Trust, Leeds, LS9 7TF, United Kingdom; Radiology.Leeds Teaching Hospitals NHS Trust, Leeds, LS9 7TF, United Kingdom; Radiology.Leeds Teaching Hospitals NHS Trust, Leeds, LS9 7TF, United Kingdom; Radiology.Leeds Teaching Hospitals NHS Trust, Leeds, LS9 7TF, United Kingdom; NHS Greater Glasgow and Clyde, Glasgow, G3 8SJ, United Kingdom; Health Tech Innovation and Translation Lab, University of Glasgow, Glasgow, G3 8SJ, United Kingdom; Newton’s Tree, London, W1B 1NT, United Kingdom

**Keywords:** artificial intelligence, radiology AI, post-deployment monitoring, automation bias, clinical governance, federated monitoring

## Abstract

**Objectives:**

To evaluate feasibility of a federated AI monitoring service (FAMOS) for post-deployment surveillance of third-party AI applications used in chest X-ray (CXR) interpretation.

**Methods:**

FAMOS was deployed at 2 NHS Trusts using a federated architecture enabling local data processing while maintaining data governance compliance. De-identified CXRs from patients aged >18 years were retrospectively identified, along with relevant patient attributes (age, sex, inpatient status, image orientation, season, artefact). Chest X-rays were processed by 2 AI applications to simulate real-world deployment. FAMOS analyzed input data, AI inference values, and longitudinal human-AI agreement as proxy indicators of data, prediction, and behavioral drift. Input monitoring used image feature embeddings analyzed with principal component analysis and Hotelling’s *T*² statistics, while risk-adjusted cumulative sum charts were applied to sequentially monitor AI inference values. human-AI agreement was evaluated at 10 time points over 3 months.

**Results:**

Input monitoring identified a small proportion of outlier examinations, predominantly associated with modifiable image quality issues. Artificial intelligence inference values remained stable across most findings for both vendors, with limited drift events detected. Human-AI agreement patterns differed between sites, remaining stable at 1 site while increasing over time at another, suggesting evolving automation bias.

**Conclusions:**

Real-time, federated monitoring of deployed radiology AI systems is technically and operationally feasible within clinical environments. Multi-domain platform-based monitoring provides scalable, independent oversight and may function as an early-warning system supporting identification of emerging risks following deployment.

**Advances in knowledge:**

This study introduces a federated, multi-domain monitoring framework integrating sociotechnical indicators for continuous post-deployment surveillance of clinical radiology AI tools.

## Introduction

As the number of radiology artificial intelligence (AI) applications approved for clinical use continues to grow and adoption becomes more widespread,^1^ ensuring their long-term safety, reliability, and effectiveness in real-world clinical settings remains a critical challenge.^2^^,^^3^

Despite the recognized need for robust and continuous post-deployment monitoring,^4–8^ current approaches remain underdeveloped.^9^ As sociotechnical systems, AI tools both influence and are influenced by the complex healthcare environments in which they are deployed.^10^ Effective monitoring must therefore look beyond pre-deployment technical performance metrics, to account for the complexities of real-world deployment, such as evolving patient populations, changing disease prevalence, clinical workflows, and human factors, all of which may introduce unforeseen risks.^2^^,^^11^

In practice, monitoring frequently relies on manual, reactive, and ad hoc methods, including informal end-user feedback, retrospective audits, or adverse event reporting.^12^ These approaches are inconsistent, resource-intensive, and often depend on access to ground-truth labels. They are typically tailored to specific workflows or use cases, limiting suitability for scalable, system-wide oversight.^13^ Consequently, they may fail to detect emerging issues promptly or capture the full range of safety risks arising during routine clinical use. These limitations pose risks to patient safety, may amplify disparities and undermine trust in AI technologies.

These approaches are also poorly equipped to identify gradual performance drift,^2^^,^^14^ which can often go undetected in the absence of structured surveillance.^15^ The causes of drift are multifactorial,^11^ including changes in input data (covariate drift)^16^^,^^17^—for example, following scanner replacement, acquisition protocol modifications, or changes in disease prevalence—shifts in prediction stability^18^ and clinician cognitive biases^19^ that may lead to over- or under-reliance on AI outputs. Detecting early signs of drift requires systematic monitoring across socio-technical factors.

However, many healthcare institutions lack the digital infrastructure and specialist workforce expertise^20–24^ required to implement such monitoring holistically. Establishing and maintaining monitoring systems at scale also entails sustained financial investment.^22^ These challenges are compounded by lack of standardized frameworks and limited practical guidance for real-world implementation including which metrics to track and how monitoring should be operationalized.^9^^,^^25^ Unclear expectations and responsibilities among key stakeholders further complicate effective oversight.^26–28^ Collectively, these technical, organizational and governance gaps underscore the need for a proactive, systematic, and scalable monitoring approach that can identify emerging risks and inform timely corrective actions to support safe and responsible integration of AI into clinical practice.

This feasibility study developed and implemented a federated pipeline for real-time monitoring of radiology AI tools, whereby data were processed locally at each site and only aggregated, non-identifiable monitoring outputs were shared centrally. The Federated AI Monitoring Service (FAMOS) is an automated model-agnostic privacy-preserving platform which tracks changes in input data quality, AI prediction distributions, and human-AI concordance, providing real-time analytics on trends that may indicate emerging clinical risk. The platform is designed to function as an early warning system, enabling sites to respond to potential issues at the point of care, before they translate into patient harm.

The study aim was to assess feasibility of FAMOS to detect changes in real-time during monitoring third-party AI applications used in chest X-ray (CXR) interpretation.

## Methods

### Study design and setting

This was a retrospective, multi-center, observational in silico evaluation study simulating the post-deployment monitoring of 2 commercially available third-party CXR AI applications across 2 large tertiary NHS institutions: site 1 and site 2 (Figure 1). Monitoring multiple hospitals at 2 large NHS Trusts enabled assessment of generalizability across different clinical settings and patient populations.

**Figure 1:**
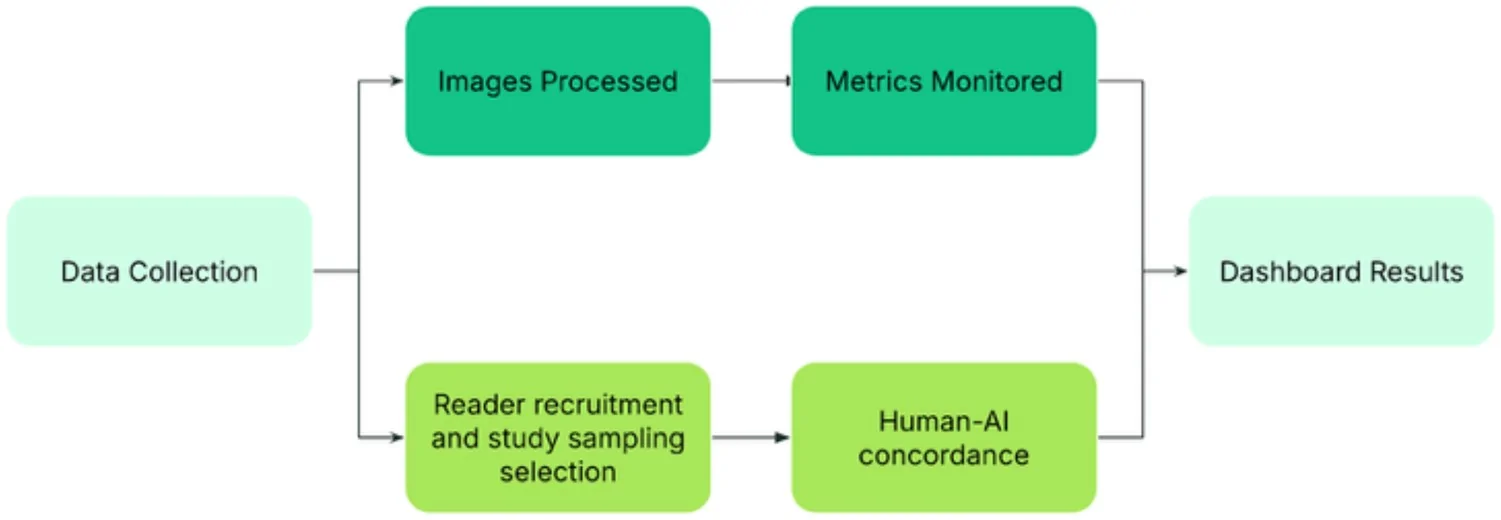
Study design illustrating the simulated monitoring pipeline.

### Data and cohort selection

A retrospective cohort of CXRs was assembled independently at each site using the local Picture Archiving and Communication System (PACS) servers, screening against inclusion criteria below. The sampling criteria were selected to ensure representation across key patient and image-level characteristics that were hypothesized to influence AI performance. The predefined stratification variables included patient sex (male vs female), patient age (<60 vs ≥60 years), patient status (inpatient vs outpatient), imaging projection (posterior-anterior vs other), seasonality (time of year of image acquisition), and presence of image artifact through visual inspection by local radiology staff.

### Inclusion criteria

Adult patients (age ≥18 years) with a CXR acquired prior to September 2024, where a corresponding radiology report was available. Studies were required to have complete metadata for the required variables as listed above.

### Sample size

A stratified sampling method was used, with strata defined a priori by a steering committee based on feasibility of collection. For each stratum, a minimum of 383 CXRs was required, which was rounded up to 400 CXRs per stratum to account for potential exclusions due to missing metadata or reports. A single CXR could contribute to more than 1 stratum. This sample size was calculated using a standard single-proportion formula with finite population correction, a confidence level of 95% was assumed (*Z* = 1.96). In the absence of prior estimates, the proportion *p* was conservatively set to 0.5 to maximize variance.

For longitudinal assessment of human-AI concordance over time, the required sample size was calculated at 18 CXR interpretations per time point to provide 80% power to detect a medium effect size (0.3) in agreement rate, accounting for autocorrelation between measurements. As the study included 10 time points, this corresponded to a total of 180 interpretations per reader across the study period.

### Ethics and data handling

Ethical approval to use de-identified, routinely collected clinical data from electronic health records was granted by the National Health Service (NHS) Health Research Authority at each site. Delegated research ethics approval was granted for linkage to NHS patient data by the Local Privacy and Advisory Committee at NHS Greater Glasgow and Clyde.

Collected data were stored securely in the Newton’s Tree sandbox which hosts a local database on the hospital network, ensuring compliance with institutional data protection policies. CXRs were processed locally at each site. Only aggregated, non-identifiable monitoring outputs were shared, in alignment with the privacy-preserving goals of the study.

### AI applications

Both AI applications were used “off-the-shelf” and were not fine-tuned for local data. The objective was not to evaluate diagnostic accuracy of the included AI systems nor to compare diagnostic performance between vendors, but to evaluate whether the monitoring framework could detect changes in input data, AI prediction distribution shift, and human-AI concordance across different products. Two AI tools were included to reduce the risk that the FAMOS design was overfitted to a single vendor.

The applications were selected as representative commercially available CXR AI tools used in routine clinical workflows. Both applications were designed to identify a range of radiographic abnormalities on CXR. Each application generated automated outputs for each CXR, including secondary capture images and structured list of findings. No vendor model updates occurred during the study period.

### Platform deployment

Newton’s Tree is a vendor-neutral software platform designed to support the end-to-end lifecycle of clinical AI systems within healthcare organizations. The platform provides a unified infrastructure to identify, evaluate, deploy, and continuously monitor third-party and in-house AI applications within real-world clinical workflows. It integrates with hospital systems (eg, EHR, PACS, RIS) using standard interoperability protocols (eg, HL7, FHIR, DICOM) to enable secure data ingestion, workflow orchestration, and auditability at the level of individual patient interactions.

A core feature of the platform is its monitoring framework FAMOS, which captures and analyses signals across 3 domains: input data characteristics, model outputs and behavior, and downstream human-AI interaction. This enables reference-free, continuous performance, and safety surveillance without requiring routine ground truth labeling. The platform also provides governance tooling, including audit logs, model registries, and reporting dashboards, to support regulatory compliance, clinical safety assurance, and operational oversight of AI systems at scale.

FAMOS was deployed locally at each participating site. Both AI applications were containerized within the Newton’s Tree Platform and processed CXRs on-site within a secure sandbox environment. Monitoring outputs were visualized on a central dashboard to support continuous oversight (Figure 2).

**Figure 2:**
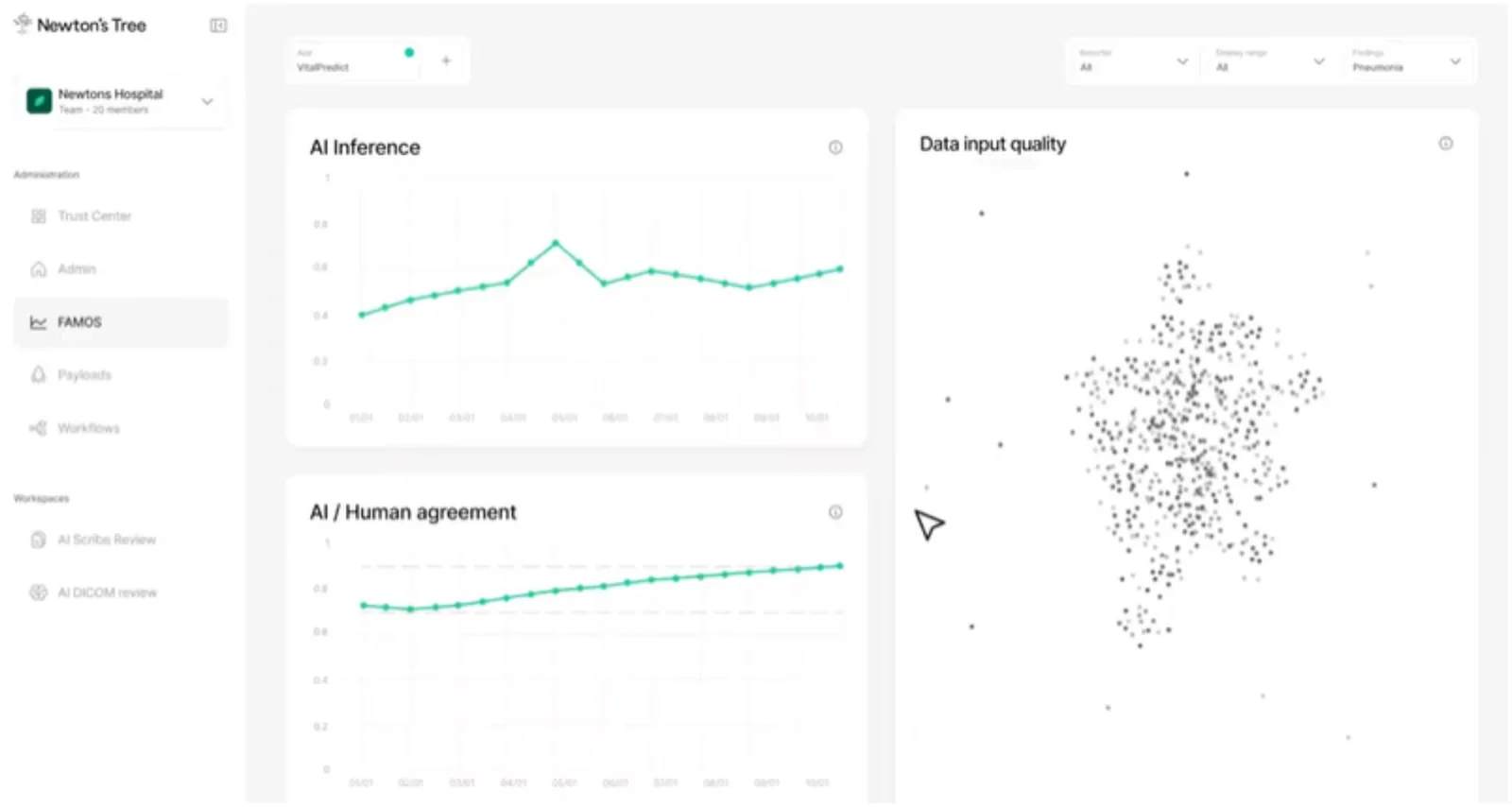
FAMOS monitoring dashboard displaying aggregated information on input data quality, AI inference distribution, and human-AI concordance.

### Monitoring workflow

Monitoring of input data and AI inference values was performed continuously during CXR processing, both serving as surrogate measures for data and prediction drift. AI inference outputs were monitored longitudinally for each deployed application using a covariate-adjusted cumulative sum (RA-CUSUM) approach. Adjustment was limited to age and sex and therefore may not fully account for all case-mix variation. Analyses were conducted separately for each AI vendor and restricted to the set of findings defined within that vendor’s output schema.

For each finding, sequential AI predictions (eg, binary classifications or probability scores) were evaluated over time to detect systematic deviations from expected behavior. RA-CUSUM charts were constructed to identify directional drift, with predefined control limits and thresholds used to signal potential over-finding (increased positive inference rates) or under-finding (decreased positive inference rates). No harmonization of findings across vendors was performed.

Raw platform data underpinning the input data quality and AI inference monitoring modules were retained for 30 days within the deployed environment. Study data for site 2 were extracted after this retention window had elapsed precluding re-analysis; consequently, these 2 monitoring domains are reported for site 1 only.

### Reader workflow

To longitudinally assess potential automation bias, case-level agreement between readers and AI outputs was evaluated at 10 time points over 3 months using a purpose-built AI review tool within the platform.

Four readers were recruited at each site, with 2 readers assigned to review the outputs from each AI application. At both sites, readers were credentialed to independently issue CXR reports. Site 1 readers were experienced reporting radiographers. Site 2 readers were senior post-fellowship exams clinical radiology registrars.

The sample size calculation for a time series analysis will depend on different factors than for a typical cross-sectional or paired comparison study. In this case, the goal is to monitor reader performance over multiple time points and detect changes in concordance between readers and AI over time. A general formula for repeated measures analysis was used with the following values: a medium effect size *d*: 0.3; autocorrelation *P*: .5; 10 time points; power: 80%; margin of error: 5%. The estimated sample size required per time point is 18 CXR interpretations. Over 10 sessions, this would total 180 interpretations per reader.

This sample size will provide enough power (80%) to detect a medium effect size (0.3) in the change of concordance with AI outputs over time, while accounting for autocorrelation between measurements.

A total of 180 CXRs were randomly sampled from the main retrospective dataset at each site, and all readers at a given site reviewed the same set of 180 CXRs. At each time point, each reader reviewed 18 cases; however, cases reviewed at a given time point were not identical across readers.

Readers did not have access to the original clinical radiology reports. Blinding to the vendor was not feasible due to the presence of vendor-specific secondary capture outputs. Using the platform’s AI review tool, readers were asked to either “Accept” or “Reject” the AI output for each case. “Accept” was defined as agreement with all AI-reported findings. If the AI output was rejected, readers were required to select a reason from a predefined drop-down list (“input data incorrect,” “results incorrect,” “result poor quality”). Prior to study commencement, readers completed a training session on use of the AI review tool and criteria for accept/reject. Because acceptance required agreement with all AI-reported findings, concordance rates may be influenced by the number of findings generated by each AI application and should therefore be interpreted as workflow-level rather than finding-level measures.

### Outcomes

The primary outcomes were (1) feasibility of platform deployment and monitoring of multiple AI applications across 2 NHS sites and (2) the ability of the monitoring framework to detect temporal changes in input data, AI inference values, and human-AI agreement following deployment. Secondary outcomes included exploration of longitudinal trends in reader behavior across sessions and between sites.

### Statistical analysis

#### Input data quality

Image embeddings were extracted from CXRs using the Microsoft RAD-DINO model.^29^ To reduce the high dimensionality of the dataset, principal component analysis (PCA) was applied to the embedding vectors.^30^^,^^31^ The number of retained components was determined using parallel analysis,^32^ retaining components whose eigenvalues exceeded those expected by chance. Standardized PCA scores were subsequently used for outlier detection using Hotelling’s *T*² statistic.^33^ To avoid reliance on theoretical distributional assumptions, an empirical threshold for *T*² was derived using 1000 bootstrap samples. For each bootstrap iteration, the bootstrap sample of the standardized PCA scores was drawn with replacement, and the corresponding mean vector and covariance matrix were recalculated. Hotelling’s *T*² values were then computed for all observations in that bootstrap sample, and the 90th percentile of these *T*^2^ values was retained as the bootstrap statistic. The final outlier threshold was defined as the 95th percentile of the 1000 bootstrap-derived statistics. Observations with *T*² values exceeding this threshold were classified as outliers. All analyses were conducted in R (version 4.4.2) using functions from *paran*, *psych*, and *ggplot2* packages for parallel analysis, PCA visualization, and plotting. Hotelling’s *T*² statistics and the bootstrap-based threshold were computed using custom R functions written specifically for this analysis, allowing full control over the statistical procedure and avoiding assumptions imposed by prepackaged routines.

#### AI inference values

For each imaging finding, a separate predictive model was developed via logistic regression to estimate the expected probability of positive AI output. These models were fitted using only baseline cases and included routinely available covariates, eg, patient age and sex. The resulting predicted probabilities represented the covariate-adjusted likelihood of each finding for each scan and served as the expected benchmark against which observed AI outputs were compared over time.

To monitor temporal changes in AI behavior, covariate-adjusted cumulative sum (RA-CUSUM) charts were constructed for every finding.^34^^,^^35^ RA-CUSUMs accumulate sequential log-likelihood ratios comparing the observed AI output (positive/negative) with the expected probability derived from the risk model. This approach enables early detection of systematic deviations in AI inference values while accounting for case mix differences.

Two types of potential drifts were evaluated. Overfinding was represented as a 2-fold increase in the odds of a positive output (odds multiplier = 2.0). Underfinding was represented as halving of the odds of a positive output (odds multiplier = 0.5). These values of multiplicative odds shifts defined the alternative hypotheses against which the RA-CUSUM charts accumulate the evidence. Because the analytical formulas for RA-CUSUM control limits are not available for risk adjusted models with heterogeneous probabilities, decision thresholds were derived through Monte-Carlo simulations. For each finding, 5000 simulated trajectories were generated under the null hypothesis of stable performance (odds multiplier = 1). For both overfinding and underfinding scenarios, the control limit *h* was chosen such that the probability of the false alarm was approximately 5% (*α*  =  .05). This ensured that any observed signal reflected a statistically meaningful deviation rather than random fluctuation. Observed RA-CUSUM trajectories were then computed for the full study period. A signal was recorded when the RA-CUSUM curve crossed the corresponding control limit: the upper limit for overfinding (OR = 2) and the lower limit for underfinding (OR = 0.5). The timing and direction of each signal indicated when the AI system began behaving as if the odds of a positive output had shifted relative to the covariate-adjusted expectation. All drift analyses were conducted in R (version 4.4.2). Logistic regression models were fitted using base R functions, and RA-CUSUM charts—including simulation-based threshold estimation—were generated using *qcc* and *racusum* quality-control routines implemented in R. Separate analyses were performed for both AI systems, and RA-CUSUM charts were produced for all findings included in the study datasets.

#### Human-AI concordance

Reader-level data were structured to enable longitudinal assessment of how readers’ agreement with AI outputs evolved over time. For each reader, all reviewed cases were ordered chronologically using the recorded time of review, ensuring that temporal patterns in acceptance of AI suggestions could be examined consistently across applications, workflows, and reading sessions. Overall concordance behavior was first described by summarizing mean agreement rates by reader, AI application, and session, providing an initial view of how frequently readers accepted the AI output and whether broad shifts in patterns of acceptance behavior emerged as experience accumulated.

To formally evaluate whether agreement changed over time, the probability of accepting the AI output was modeled using mixed-effects logistic regression. The outcome variable was the binary indicator of concordance between the reader and the AI system. Because multiple observations were contributed by each reader their identity was included as a random intercept to account for clustering and to capture differences in baseline agreement levels. Reading session number was treated as a continuous fixed effect representing the temporal dimension of exposure to AI. This modeling strategy provided an adjusted estimate of how agreement changed with each additional session, while controlling for between-reader variability. The model estimated the baseline log-odds of agreement and the change in these log-odds across sessions. A positive and statistically significant time effect indicated that readers became increasingly likely to accept the AI output as sessions progressed, consistent with the development of automation bias. A negative effect suggested decreasing reliance on AI recommendations over time. All results were reported on the logit scale, reflecting the linear predictor of the mixed-effects model.

To evaluate temporal changes in AI-agreement behavior, cumulative sum (CUSUM) analysis was also applied to each reader’s sequence of decisions. A baseline expected agreement rate was first calculated across all readers; this value served as the reference level against which individual behavior was compared. For each reader, cases were ordered by review time and the CUSUM statistic was constructed by cumulatively summing the deviation between the observed agreement outcome and the baseline rate. This caused the trajectory to drift upward when agreement exceeded expectations and downward when it fell below.

To distinguish meaningful behavioral change from normal variation, curved control limits were added to readers’ CUSUM chart. CUSUM trajectories were compared against control limits, and threshold crossings were interpreted as evidence of a sustained shift in agreement behavior over time that was unlikely to occur due to chance alone. This approach allowed identification of readers whose reliance on AI increased or decreased over time in a manner consistent with behavioral drift, including potential automation bias. Human-AI concordance analyses were conducted in Stata (version 19).

## Results

### Data cohort and selection

A total of 898 and 564 CXRs were processed through the platform at site 1 and site 2, respectively. The mean latency between image ingestion and dashboard visualization was 20 seconds.

### Monitoring workflow

#### Input data quality (site 1)

Input data quality analysis was available for site 1 only, using image embeddings derived from 898 scans. Parallel analysis supported retention of 27 principal components, explaining 88% of the total variance. Using Hotelling’s *T*² statistic multivariate outlier detection, 78 cases exceeded the bootstrap-derived threshold (*T*² = 53.276) (Figure 3). Of the flagged outliers, 90% reflected true image quality issues, such as suboptimal patient positioning (43/66), implants/medical devices (16/66), and image artifacts (12/66). Categories were not mutually exclusive and individual cases could contribute to more than one category.

**Figure 3:**
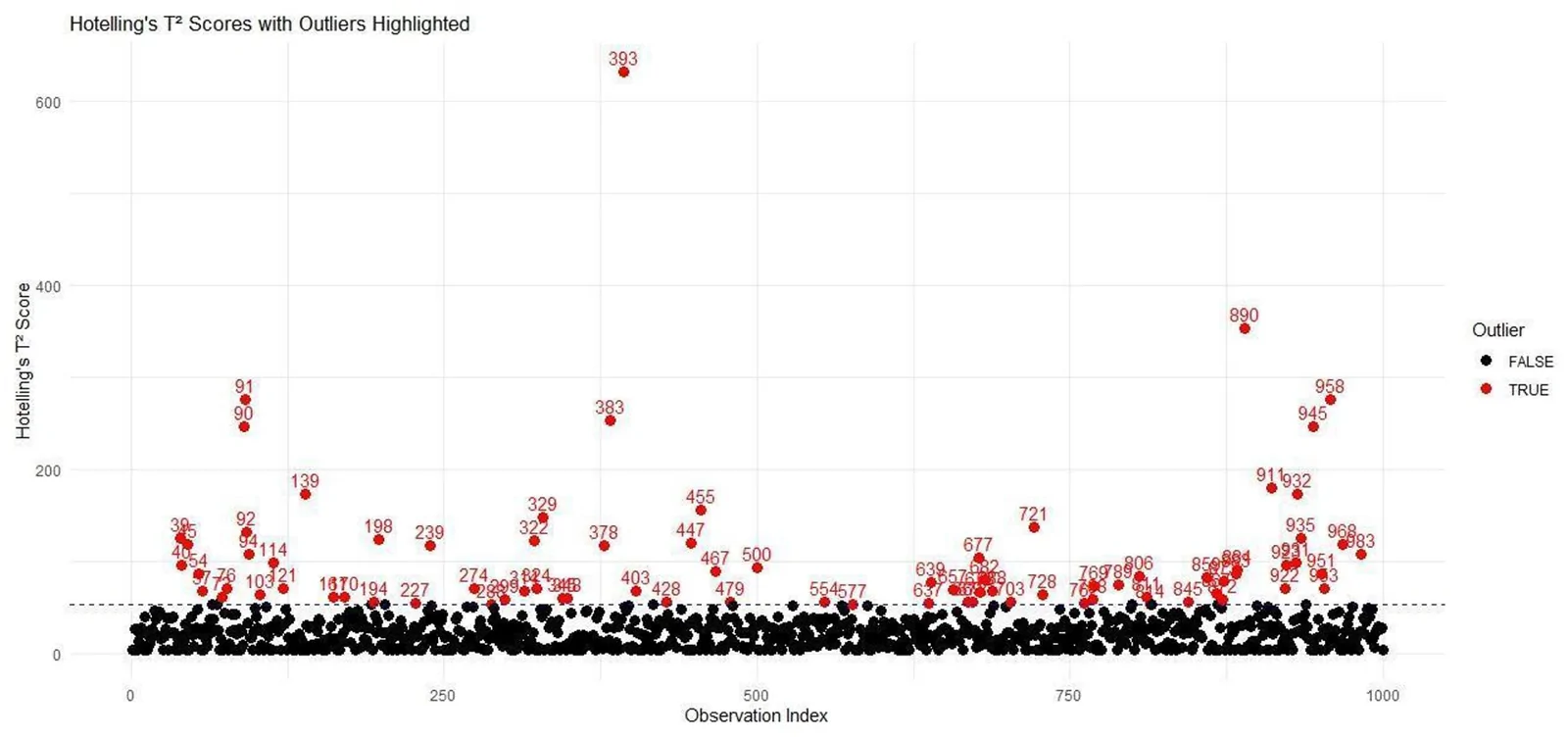
Hotelling’s *T*² scores for all observations at site 1, with outliers (*T*² > 53.276) labelled with their case ID.

#### AI inference values (site 1)

AI inference value analyses were available for site 1 only, as the corresponding site 2 raw monitoring data were no longer retrievable after expiry of the platform retention window. RA-CUSUM monitoring was applied separately to both AI applications across all findings. Analyses were performed within each vendor’s own finding set to assess prediction drift. No direct comparison of vendor performance was conducted.

Vendor 1 demonstrated stable AI inference values for most findings, with drift signals observed for diffuse upper airspace opacity (over-finding) and for aortic arch calcification and unfolded aorta (under-finding). Vendor 2 showed a similar overall pattern of stability, with a single over-finding demonstrated for mediastinal mass (Figure 4A-D).

**Figure 4:**
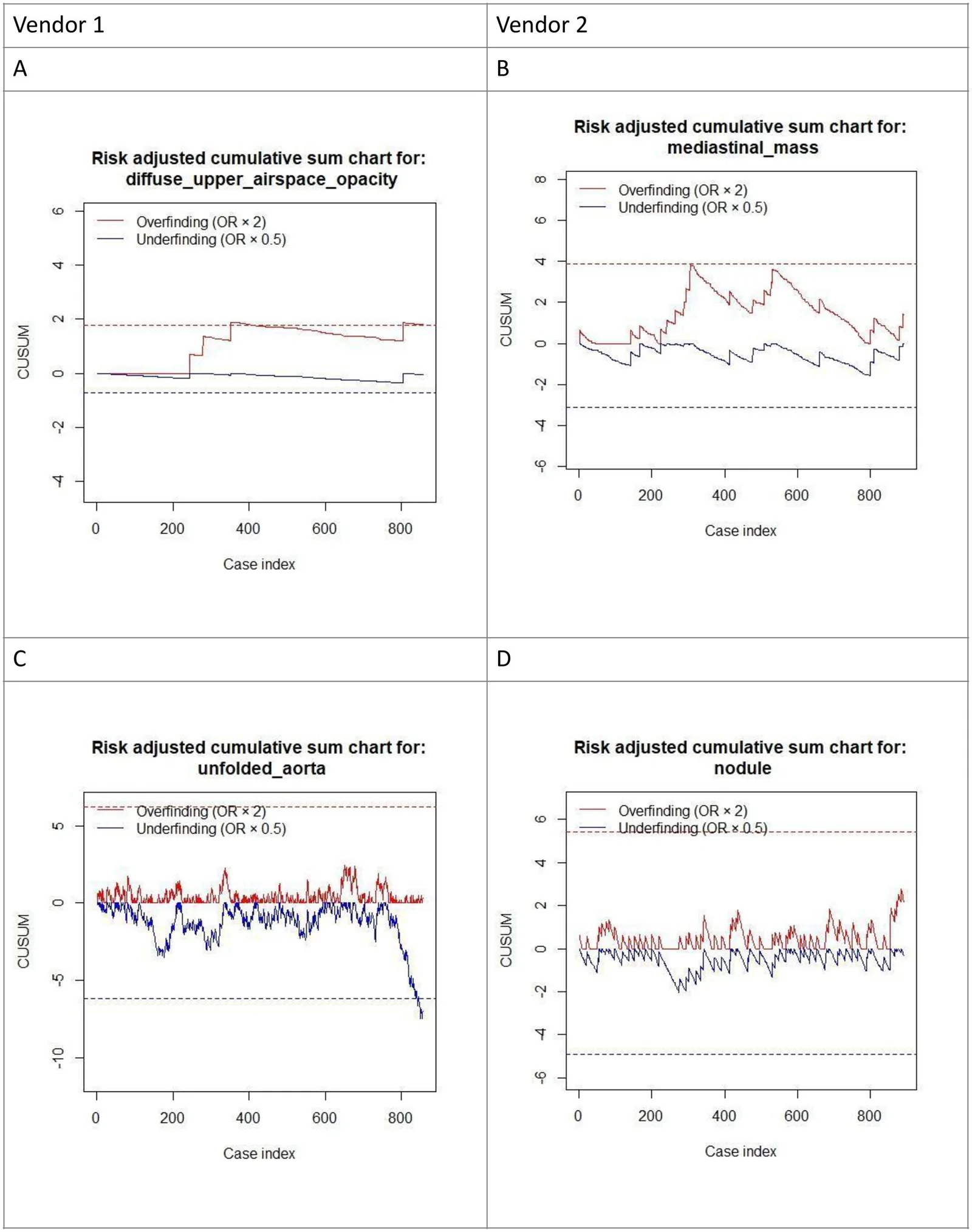
A-D presents the RA-CUSUM charts for selected findings evaluated with vendor 1 and vendor 2. Each plot includes control limits and directional drift thresholds.

#### Human-AI concordance

At site 1, concordance rates across readers and sessions ranged from 0.44 to 0.89, with no consistent increase over time. Mixed-effects logistic regression findings were consistent with these trends. Agreement did not increase across sessions (session coefficient −0.010). Baseline agreement was high (72% concordance at session 0) while between-reader variation was limited (random intercept variance 0.020). CUSUM analysis demonstrated that all trajectories remained within control limits across the 180 reviewed cases per reader, with no sustained upward drift in agreement (Figure 5; Table 1).

**Figure 5:**
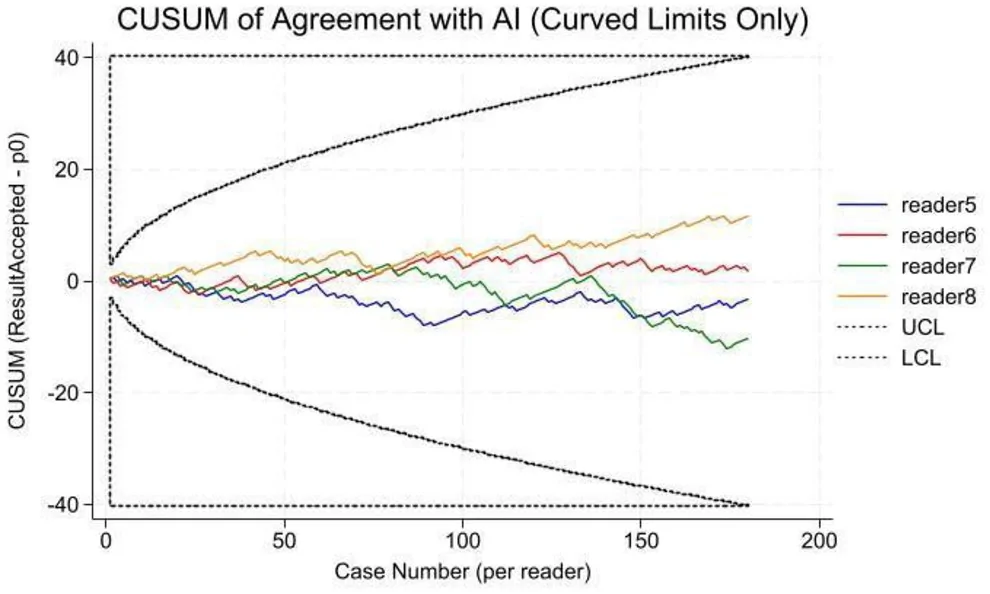
Site 1: CUSUM trajectories of AI agreement, with curved control limits. Each line represents the cumulative deviation from baseline agreement for 1 reader. Reader 5-8 represents the 4 anonymous readers from site 1.

In site 2, concordance rates across readers and sessions ranged from 0.56 to 1.00. The initial graphical examination of means revealed a slight general upward trend: as session number increased, the mean concordance values tend to rise too. This pattern suggested that in later sessions readers show greater agreement with AI outputs, especially in categories 6-10. The results of regression analysis (Table 2) revealed that session coefficient was positive and statistically significant ($\beta_{1}=0.074$, *P = .*020), indicating that the log-odds of agreement increased with each additional session. The observed increase in agreement over time indicates increasing human-AI concordance. However, in the absence of ground truth, this design cannot distinguish appropriate calibration and trust in AI outputs from true automation bias. Therefore, concordance trends should be interpreted as behavioral signals warranting further investigation rather than direct evidence of automation bias.

**Table 2:** Site 2: mixed-effects logistic regression results for automation bias (site 2), presented in linear (log-odds) form.

Predictor	Coefficient (*β*)	Std. error	*z* value	*P* value	95% CI (lower, upper)
**Fixed effects**					
** Intercept (*β*₀)**	0.832	0.267	3.12	<.001	(0.310-1.356)
** Session (*β*₁)**	0.074	0.031	2.33	.020	(0.012-0.137)
**Random effects**					
** Reader-level variance ($\sigma_{u}^{2}$)**	0.144	0.127	–	–	(0.025-0.811)

The session coefficient was 0.074, meaning that the log-odds of agreement increased by 0.074 for each additional reading session.

The estimated variance of the random intercept ($\sigma_{u}^{2}=0.144$) was small, suggesting modest but non-negligible differences between readers in their baseline tendency to agree with AI. The intercept ($\beta_{0}=0.832$) was positive and significant, corresponding to a baseline probability of roughly 70% agreement at session = 0. This indicates that readers already showed a relatively high level of concordance with AI at the start, and the positive session slope (as discussed above) suggests that this tendency strengthened over time.

The CUSUM chart for the site 2 data (Figure  6) shows how each reader’s agreement with the AI changed over time relative to the expected reference level (*p*₀ = 0.70, derived from the pooled baseline agreement rate across all site 2 readers). Upward drift reflects more agreement than expected, while downward drift reflects less. The curved control limits indicate the range of variation expected under stable behavior; crossing them would signal a meaningful shift rather than random fluctuation. Reader 2 shows a clear upward trend approaching the upper limit, suggesting increasing agreement with the AI and a pattern consistent with emerging automation bias. Reader 3 shows the opposite pattern, with a downward drift indicating progressively lower agreement and increasing independence from the AI. Reader 1 remains stable around 0, showing no systematic change, and Reader 4 fluctuates without a sustained upward trend. Importantly, none of the readers crossed the control limits, meaning that although some trends are suggestive, no statistically confirmed behavioral shift occurred. Overall, the CUSUM analysis highlights heterogeneity across readers: only Reader 2 shows a trajectory consistent with automation bias, while the others remain stable or diverge from AI recommendations over time.

**Figure 6:**
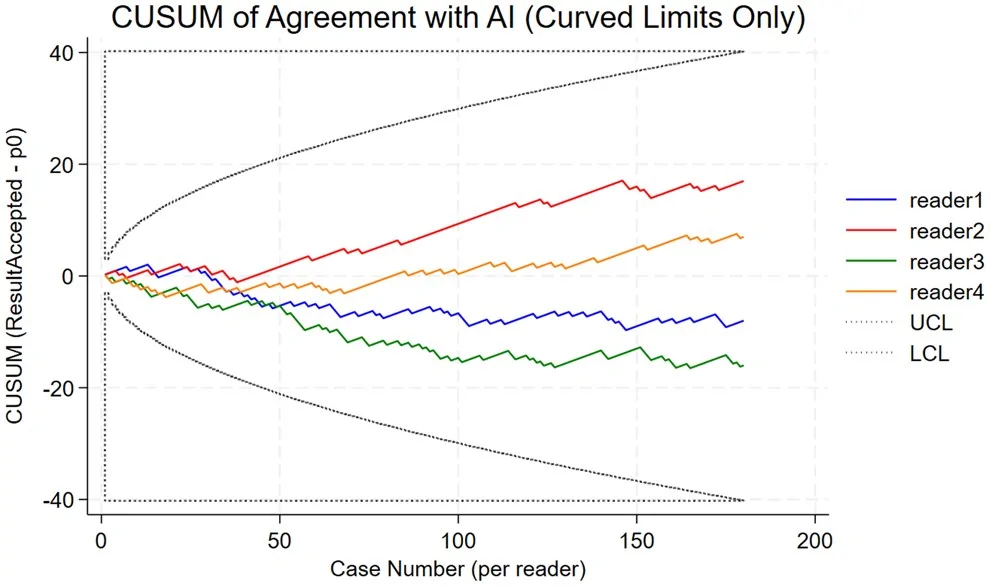
Site 2: CUSUM trajectories of AI agreement, with curved control limits. Each line represents the cumulative deviation from baseline agreement for 1 reader. Reader 5-8 represents the 4 anonymous readers from site 1.

## Discussion

In this study, a real-time, federated, automated monitoring platform for post-deployment surveillance of third-party radiology AI tools was implemented and evaluated across 2 NHS sites. The platform tracked input data, AI inference values, and longitudinal human-AI agreement as proxy measures for potential drift and automation bias, respectively. The findings demonstrate that continuous monitoring is both technically and operationally feasible within simulated clinical environments and supports the scalability of a platform-based solution integrating multiple monitoring domains with centralized oversight. The FAMOS framework represents a pragmatic strategy in which monitoring outputs function as an early-warning mechanism by prompting targeted review before issues translate into patient harm. When integrating into established clinical risk management and safety processes,^36–38^ the monitoring outputs may also enable healthcare organizations to transition from ad hoc retrospective audits and incident reporting toward more proactive risk surveillance.

An important contribution of this work is the demonstration that post-deployment monitoring can be implemented using a federated architecture. This design aligns with information governance requirements by enabling data to be processed locally while providing cross-site visibility of AI behavior without exposing patient information. It may also facilitate shared insight among healthcare providers, vendors, and regulators regarding real-world AI performance and emerging risks,^2^ helping to reduce fragmentation and siloing of monitoring information where different stakeholders often have partial visibility into AI behavior. In turn, this supports more coordinated, transparent and accountable oversight of deployed clinical AI systems.

Furthermore, the FAMOS framework complements vendor-reported monitoring metrics, which may not fully capture local deployment conditions or human-AI interaction effects and aligns with emerging recommendations advocating independent post-market surveillance of clinical AI systems.^2^ Centralized monitoring across multiple AI vendors may also streamline operational costs by avoiding the need for healthcare organizations to develop separate in-house monitoring solutions for each AI application.

### Relation to prior work

A growing body of evidence has demonstrated that clinical AI tools are susceptible to covariate and concept drift, leading to temporal performance degradation,^2^^,^^14^ reinforcing the need for robust post-deployment monitoring strategies. Several studies have proposed technical approaches for detecting drift in clinical AI tools, typically focusing on individual aspects of the monitoring problem.

Input-level drift has been monitored using autoencoder-derived image representations and model-output-based drift detection experiments.^17^ Roschewitz et al.^16^ introduced unsupervised prediction alignment, which detects image acquisition shifts indirectly through changes in prediction distributions. Other approaches combine supervised and unsupervised feature representations with geometric distance metrics and statistical process control techniques, including CUSUM methods to identify out-of-distribution inputs.^39^ Prathapan et al.^40^ similarly applied CUSUM control charts for temporal monitoring of AI performance. Multi-modal monitoring frameworks such as CheXstray^41^ integrate DICOM metadata, model-predicted probabilities, and latent image representations derived from variational autoencoders, comparing features with reference datasets within sliding temporal windows and standardizing these measures into a unified drift metric.

A small number of recent reports have described operational implementations of post-deployment monitoring frameworks in clinical radiology practice. Cook et al.^42^ reported the Mayo Clinic experience, incorporating daily, weekly, and annual monitoring of utilization, failure modes, data drift, and end-user feedback using automated alerts and dashboards.

Keyes et al.^43^ developed a monitoring framework at Stanford focusing on system integrity, performance, and impact monitoring embedded within formal governance pathways. Dean et al.^44^ reported a longitudinal monitoring framework at Unilabs designed to assess both technical reliability and reader interaction with deployed AI tools.

The FAMOS monitoring framework aligns with and extends this evolving body of work by combining complementary monitoring domains spanning input data, AI inference and human-AI concordance, thereby supporting a system-level view of the clinical environment in which AI operates. This reflects a socio-technical perspective in which safe clinical AI use emerges from interactions between data, the AI model, and users.^45^

The recently published LungIMPACT randomized controlled trial further illustrates that CXR AI can be operationalized prospectively and at scale within NHS diagnostic pathways, evaluating more than 90 000 chest X-rays across multiple sites.^46^ In that study, the principal focus was pathway impact from AI-supported prioritization, whereas the present study addresses a complementary question: how deployed AI systems can be monitored continuously for changes in input characteristics, inference behavior, and human-AI concordance once operational. Together, these studies suggest that future real-world evaluations should combine pathway and outcome endpoints with structured post-deployment monitoring.

### Clinical relevance of monitoring findings

Input data monitoring identified a small subset of scans with atypical image characteristics (“outliers”), the majority corresponding to true image quality issues such as positioning errors, artifacts, or medical devices. Detection of these modifiable acquisition factors is clinically relevant because variability in imaging inputs represents a recognized precursor to downstream AI performance alteration.^16^^,^^17^ Early identification enables targeted investigation or reacquisition before AI behavior is affected or even filtering data out, illustrating the value of this monitoring method as a scalable quality control mechanism within routine radiology workflows.

Monitoring of AI inference values using CUSUM demonstrated stability across most findings over the observational period. The limited number of drift events observed suggests that sequential statistical monitoring can provide clear triggers for investigation while reducing the risk of excessive notifications that could contribute to alert fatigue. This balance is operationally important, as monitoring systems must detect meaningful deviations while remaining manageable in clinical environments. Furthermore, this approach does not rely on contemporaneous ground-truth labels, which are often unavailable in real-world settings, supporting the use of proxy measures of performance stability.

Human-AI agreement monitoring revealed heterogenous patterns across sites. Agreement remained stable at site 1 but increased at site 2 over the monitoring period. The difference may partly reflect contextual factors, including reader experience and workload. Junior clinicians at site 2 conducted reviews alongside routine clinical duties and on-call commitments, whereas predominantly experienced reporting radiographers participated at site 1 and reviewed cases during dedicated sessions. Automation bias refers to the tendency for users to over-rely on automation at the expense of independent judgment.^47^ A radiology study conducted by Dratsch et al.^19^ demonstrated that AI recommendations can influence reader interpretation behavior. Monitoring longitudinal human-AI concordance trends therefore provides insight into user behavior following clinical deployment and enables detection of behavioral drift. These findings highlight the importance of incorporating human factors alongside technical monitoring to support patient safety.^48^

### Strengths

To our knowledge, this study is among the first to operationalize federated monitoring of input data, AI predictions, and human-AI agreement within simulated clinical environments. The strengths of this work include integration of multiple monitoring domains within a single pipeline, reflecting real-world determinants of clinical AI safety. The federated architecture enabled cross-site monitoring while maintaining data governance compliance. The monitoring approach does not rely on continuous ground-truth labeling which is often impractical and resource-intensive in clinical settings. Finally, the framework is modular and extensible, allowing additional monitoring components to be incorporated as regulatory guidance evolves.

### Limitations

This study has several limitations. The evaluation was retrospective and conducted in silico; longer prospective deployment is required to assess performance of the monitoring pipeline under live clinical conditions and to evaluate downstream clinical impact. Generalizability is limited by inclusion of 2 sites, two-third party AI applications, a relatively small number of cases and readers, and the absence of consultant-level participants. Estimates of between-reader heterogeneity should be interpreted cautiously because only 4 readers were included per site, resulting in imprecise variance estimates and wide confidence intervals. Case difficulty was not stratified in the human-AI agreement part of the study, which may have influenced observed concordance patterns.

Secondary analyses of input data and AI inference distributions were restricted to 1 site because the corresponding site 2 raw monitoring data were no longer available after expiry of the platform retention window, limiting cross-site comparison of deviations identified through outlier detection and CUSUM monitoring. Multiple RA-CUSUM analyses were performed across findings, vendors and drift directions. While control limits were derived independently for each analysis, no formal multiplicity adjustment was applied and therefore some detected signals may represent chance findings.

The loss of site 2 monitoring data represents an important operational finding. Future deployments should implement longer retention periods, automated extraction schedules, or preservation of monitoring outputs within the clinical risk management process to ensure complete longitudinal surveillance datasets.

Finally, while monitoring outputs identified potential outliers, corrective actions were not evaluated as this was beyond the scope of this feasibility study.

### Future work

Future work should evaluate prospective deployment of the FAMOS platform across additional sites and imaging modalities, define and validate escalation thresholds, and assess how monitoring outputs integrate into local clinical risk management processes and regulatory reporting pathways. An important operational lesson from this study relates to monitoring data retention. Continuous monitoring systems require governance arrangements that ensure monitoring outputs are retained for a period sufficient to support audit, investigation, and retrospective analysis. Future deployments should define retention requirements prospectively, aligned to local clinical governance and clinical risk management processes, and implement automated extraction or archiving procedures to minimize loss of monitoring information.

Future studies should also include prospective evaluation of implementation costs, staffing requirements, return on investment, and broader patient and pathway benefits, as well as more systematic assessment of technology acceptance, human factors, and downstream diagnostic and health-economic impact.

**Table 1:** Site 1: mixed-effects logistic regression results for automation bias (site 1), presented in linear (log-odds) form.

Predictor	Coefficient (*β*)	Std. error	*z* value	*P* value	95% CI (lower, upper)
**Fixed effects**					
** Intercept (*β*₀)**	0.941	0.192	4.91	<.001	(0.565 to 1.317)
** Session (*β*₁)**	−0.010	0.029	−0.36	.721	(−0.066 to 0.046)
**Random effects**					
** Reader-level variance ($\sigma_{u}^{2}$)**	0.020	0.034	–	–	(0.001 to 0.543)

## Conclusion

A federated framework for real-time monitoring of third-party radiology AI applications, that tracks input data quality, AI inference distribution, and human-AI concordance metrics is feasible. This approach can function as an early warning system, enabling timely identification of emerging risks and strengthening clinical risk management processes, providing a practical framework toward safe, responsible, and scalable oversight of AI in clinical care.

## Author contributions

Aysha Luis (Formal analysis, Investigation, Methodology, Project administration, Visualization, Writing – original draft, Writing – review & editing), Andrew Scarsbrook (Conceptualization, Funding acquisition, Resources, Supervision, Writing – review & editing), Mariusz Grzeda (Formal analysis, Methodology, Visualization, Writing – original draft, Writing – review & editing), Jesus Perdomo Lampignano (Data curation, Investigation, Project administration, Writing – review & editing), Matt Clark (Data curation, Investigation, Project administration), Bob Wheller (Project administration, Resources, Software), Jack Baldwin (Data curation, Investigation), James Cairns (Data curation, Investigation, Validation, Writing – review & editing), Simran Dhesi (Investigation, Validation, Writing – review & editing), Mark Hall (Data curation, Investigation, Resources, Supervision, Writing – review & editing), David J. Lowe (Conceptualization, Funding acquisition, Resources, Supervision, Writing – review & editing), Haris Shuaib (Conceptualization, Funding acquisition, Methodology, Project administration, Resources, Software, Supervision, Writing – review & editing).

## Data Availability

The data underlying this article cannot be shared publicly for reasons of patient privacy and information governance; images, metadata, AI outputs and reader decisions remained within each NHS organisation's secure environment under the approvals granted.
